# Berberine modulates deacetylation of PPARγ to promote adipose tissue remodeling and thermogenesis via AMPK/SIRT1 pathway

**DOI:** 10.7150/ijbs.62556

**Published:** 2021-07-25

**Authors:** Yingxi Xu, Tianhao Yu, Guojing Ma, Lixia Zheng, Xuehan Jiang, Fan Yang, Zhuo Wang, Na Li, Zheng He, Xiaoyu Song, Deliang Wen, Juan Kong, Yang Yu, Liu Cao

**Affiliations:** 1 Department of Clinical Nutrition, Shengjing Hospital of China Medical University, Shenyang, 110004, China.; 2 College of Basic Medical Science, Institute of Translational Medicine, Key Laboratory of Medical Cell Biology, Ministry of Education, Key Laboratory of Liaoning Province, China Medical University, Shenyang, Liaoning Province, P.R. China, 110122.; 3 Institute of Health Sciences, China Medical University, Shenyang 110122, Liaoning, China; 4 The VIP Department, School and Hospital of Stomatology, China Medical University, Liaoning Provincial Key Laboratory of Oral Diseases, Shenyang, 110002, China.; 5 Department of Radiation Oncology, The First Affiliated Hospital of China Medical University, Shenyang, 110001, China.

**Keywords:** Berberine, Adipose tissue, Deacetylation, PPARγ, AMPK/SIRT1 axis

## Abstract

Pharmacological stimulation of adipose tissue remodeling and thermogenesis to increase energy expenditure is expected to be a viable therapeutic strategy for obesity. Berberine has been reported to have pharmacological activity in adipose tissue to anti-obesity, while the mechanism remains unclear. Here, we observed that berberine significantly reduced the body weight and insulin resistance of high-fat diet mice by promoting the distribution of brown adipose tissue and thermogenesis. We have further demonstrated that berberine activated energy metabolic sensing pathway AMPK/SIRT1 axis to increase the level of PPARγ deacetylation, which leads to promoting adipose tissue remodeling and increasing the expression of the thermogenic protein UCP-1. These findings suggest that berberine that enhances the AMPK/SIRT1 pathway can act as a selective PPARγ activator to promote adipose tissue remodeling and thermogenesis. This study proposes a new mechanism for the regulation of berberine in adipose tissue and offers a great prospect for berberine in obesity treatment

## Introduction

Growing evidence indicates that obesity can induce disorders at hormonal, inflammatory, and endothelial levels^1^, which increases the risk of many chronic diseases, such as type 2 diabetes^2^, metabolic syndrome^3^, cardiovascular diseases^4^, stroke^5^, and cancer^6^. Maintaining energy balance is the fundamental way to overcome obesity and it mainly includes reducing nutrient intake and increasing energy expenditure. With the discovery of brown adipose tissue (BAT) in adults, the metabolizing function of BAT has attracted more attention in the field of obesity treatment^7^. Compared to white adipose tissue (WAT) that is in charge of energy storage, BAT composed of multilocular lipid droplets prefers to combust energy through non-shivering thermogenesis mediated by mitochondrial uncoupling protein 1 (UCP1)^8^-^11^. In addition to the classical thermogenic way in BAT, another potential way is the formation of brown-like adipocytes with the function of thermogenesis in WAT^9^. As the limited content and activity of BAT are unlikely to offset the positive energy balance associated with excessive WAT deposition^12^, stimulating WAT browning and activating BAT thermogenesis are considered as potential therapeutic interventions for obesity.

PPARγ is a member of the nuclear receptor superfamily of ligand-induced transcription factors, and it is specifically highly expressed in adipose tissue^13^, ^14^. PPARγ can promote the expressions of brown adipocyte-specific genes and inhibit the expressions of white adipocyte-specific genes to induce brown-like phenotype in WAT^15^. However, nonselective PPARγ agonist with these unique benefits is shadowed by the risk of side effects, such as weight gain, bone loss, fluid retention, and congestive heart failure, which prevents its clinical application^16^-^18^. Therefore, increasing studies have focused on the protein modifications of PPARγ to exploit its positive effect and avoid its side effects on metabolism^19^, ^20^. PPARγ can be regulated by post-translational modifications, including phosphorylation, acetylation, sumoylation, and ubiquitination^14^, ^21^, ^22^. K268 and K293 on PPARγ are deacetylated by Sirtuin 1 (SIRT1) in a ligand-dependent manner to recruit the BAT program coactivator Prdm16, which promotes adipose tissue remodeling^21^. Under different stimulus conditions, the deacetylation of PPARγ can selectively promote the expressions of brown adipocyte-specific genes^15^. This suggests that selective deacetylated PPARγ may be a regulatory switch for the conversion of WAT to BAT, which provides a potential strategy for the treatment of obesity.

Berberine (BBR) is an active product isolated from the medicinal plant Rhizoma Coptidis, which is used in the treatment of diarrhea. It has received increasing attention for a variety of metabolic benefits in recent years^23^, ^24^. Previous studies have demonstrated that BBR plays a vital role in improving glucolipid metabolism and insulin resistance through regulating adipose tissue. BBR can inhibit WAT differentiation and accumulation, and increase adipose tissue thermogenesis, but the mechanism is not clear^25^-^28^. BBR has been proven to be an activator of AMPK, which is responsible for triggering glucose uptake, reducing liver gluconeogenesis, and improving insulin sensitivity^29^-^31^. SIRT1 has important effects on glucose homeostasis, insulin sensitivity, and adipogenic regulation via its deacetylase activity^32^. AMPK can enhance SIRT1 activity by increasing cellular NAD^+^/NADH ratio, which deacetylates downstream SIRT1 targets and modulates their activity^33^. Therefore, we speculate BBR that enhances the AMPK/SIRT1 pathway can act as a selective PPARγ activator to promote adipose tissue remodeling and thermogenesis. This study proposes a new mechanism for the regulation of BBR in adipose tissue and offers a great prospect for BBR in obesity treatment.

## Materials and Methods

### Reagents and antibodies

BBR (purity quotient > 99.8%) was obtained from Northeast Pharmaceutical Group Co. (Shenyang, China). AICAR, Compound C, and Oil Red O were purchased from Sigma-Aldrich (MO, USA). MitoTracker^®^ Red was purchased from Invitrogen (MA, USA). The kits of T-THO (total cholesterol), TG (triglyceride), and LDL-C (low-density lipoprotein cholesterol) were purchased from Nanjing Jiancheng Biotechnology Co. (Nanjing, China). Dexamethasone, 3-isobutyl-1-methylxanthine (IBMX), indomethacin, 3,3′,5-triiodo-Lthyronine (T3), and Glucose were purchased from Sigma-Aldrich (MO, USA). Recombinant human insulin was purchased from Novo Nordisk Pharmaceuticals Inc (Copenhagen, Denmark). Antibodies used including AMPK (1:1000; Cell Signaling Technology, MA, USA), Phospho-AMPK (Thr172; 1:1000; Cell Signaling Technology, MA, USA), acetyl-lysine (1:1000; Cell Signaling Technology, MA, USA), SIRT1 (1:1000; Millipore, MA, USA), PPARγ (1:200; Santa Cruz, CA, USA), UCP-1 (1:1000; Abcam, MA, USA), and β-actin (1:2000; Sigma-Aldrich, MO, USA).

### Experimental Animals

Male six-week-old C57BL/6J mice were obtained from Beijing Vital River Laboratory Animal Technology. *Sirt*1^+/-^ mice with a deletion of exon 4 as a kind gift from Cheng et al were used for mouse embryo fibroblasts (MEF) isolation^34^. All animals were housed in standard polypropylene cages, maintained in an environmentally controlled breeding room (temperature: 20 ± 2℃, humidity: 60 ± 5%, 12 h light/dark cycle), and free access to food and water. Mice were randomly divided into four groups as NCD (normal-chow diet), HF (high-fat chow diet), BBR-25, BBR-100 group. Mice in HF, BBR-25, and BBR-100 were fed by high-fat diet containing 60% calories from fat, 20% from protein, and 20% from carbohydrates (Research Diet) from 6 weeks. After 8 weeks, the HF, BBR-25, and BBR-100 group were respectively treated with 0.9% sterile saline, BBR (25 mgkg^-1^), and BBR (100 mgkg^-1^) by gavage daily between 14:00-16:00 until 20 weeks of age. All mice were allowed to continue to feed on their respective diets until the end of the study. Body weight and food intake were recorded weekly. When mice were sacrificed, the adipose tissues were dissected and weighted. Serum and adipose tissues were collected and frozen at -80℃. All animal studies were approved by the Institutional Animal Care and Use Committee at China Medical University (CMU2021007).

### Cell culture and differentiation

3T3-L1 white preadipocytes were purchased from the American Type Culture Collection. HIB1b brown preadipocytes, kindly provided by Professor Difei Wang (Shengjing Hospital of China Medical University). MEFs were established from *Sirt*1^+/-^ mated E10.5-15.5 embryos as previously described^35^. 3T3-L1 and HIB1b cells were maintained and differentiated as previously described^36^, ^37^. SIRT1 deacetylase-inactive mutant (HY) and siRNA retrovirus have been previously described^38^. shRNA against AMPK-α (shAMPK-α) lentivirus were purchased from GeneChem Company (Shanghai, China).

### Oil Red O

Cells were washed with PBS and fixed with 4% paraformaldehyde for 20 min at 37℃. After being washed with PBS twice, cells were incubated with Oil Red O for 40 min. After staining, cells were washed with PBS twice. Stained cells were observed on an inverted light microscope (Olympus, IX73).

### Histology and immunofluorescence

After mice were sacrificed, the adipose tissues were collected and fixed in 4% paraformaldehyde, then prepared and stained with hematoxylin and eosin (HE) to analyze morphological characterization. Samples were observed on a light microscope (Olympus, BX53). Immunohistochemistry staining was performed according to the standard protocol using the anti-UCP1 antibody. For immunofluorescence, adipocytes were incubated with anti-PPARγ and anti-SIRT1 antibodies at 4℃ overnight, followed by PBS wishing, then incubated with appropriate FITC goat anti-mouse and goat anti-rabbit secondary antibodies at room temperature for 1 h. Stained cells were observed on an inverted fluorescence microscope (Nikon Biostation IM-Q).

### Mitochondrial Analysis

Cells were shifted to DMEM containing 100 nM MitoTracker Red FM for 30 min at 37℃. After incubation, cells were washed with a prewarmed culture medium and observed on an inverted fluorescence microscope (Nikon Biostation IM-Q).

### Transmission electron microscopy

Cellular ultrastructure was assessed with transmission electron microscopy as previously described^39^, ^40^. Briefly, cells were fixed in 2.5% glutaraldehyde for 12 h at 4°C, followed by washing three times with PBS. Samples were dehydrated and embedded. 60-nm thin sections of the embedded samples were mounted on copper grids. Then samples were observed with the transmission electron microscope (JEOL JEM-1400, JEOL, Tokyo, Japan).

### Glucose and insulin tolerance tests

For oral glucose tolerance test (OGTT) and intraperitoneal injection of insulin tolerance test (IPITT), mice fasted for 6 h. Mice were treated with glucose (2 gkg^-1^) by gavage or recombinant human insulin (0.75 Ukg^-1^) by intraperitoneal injection. Blood glucose levels of GTT were determined using the Contour glucometer (Bayer, Leverkusen, Germany) at 0, 30, 60, 90, and 120 min. Blood glucose levels of IPITT were determined at 0, 15, 30, 60, and 120 min after injection.

### Metabolic cage parameters

For collecting metabolic cage parameters, mice were housed individually in metabolic cages at a 12 h light-dark cycle using the Promethion Metabolic Cage System (Sable Systems, USA). Mice were acclimated to the metabolic cage for 48h before recording data. The metabolic cage collected the velocity of oxygen consumption (V_O2_), carbon dioxide production (V_CO2_), and energy expenditure.

### Micro-CT

Mice scapular BAT and its peripheral WAT were scanned by Bruker SkyScan Micro-CT (SkyScan 1276, PA, USA) at a 70 kV/200 mA source voltage/current. The pixel size (resolution), rotation step, and exposure time were set up at 40.38 μm, 0.6° over 360°, and 140 ms, respectively. The micro-CT analysis was performed with the CTAN software. 3D images of the defect area were constructed using CTVol Software Version 2.6 for imaging rendering and visualization.

### Cool exposure test

Cool exposure test was performed in a temperature-controlled room at 4℃. Mice were kept in individual cages and subjected to cold exposure for 120 min. Core temperature was measured every 30 min until the end of the test.

### Western blot and immunoprecipitation

Cells were collected and resuspended in NP-40 lysis buffer. The resuspended cells were vortexed for 10 s, incubated on ice for 20 min, and centrifuged at 20,000× g for 20 min. Then lysates were used for Western blot. For immunoprecipitation (IP), primary antibody was coupled with protein A/G beads, and then immune complex was added to the cell lysates and incubated at 4°C overnight. After that, samples were washed with NP-40 lysis buffer three times. The immunoprecipitates were subjected to SDS-PAGE and Western blot.

### Real-Time PCR analysis

The total RNA was isolated from cells or adipose tissues using an RNeasy Plus Mini Kit (Qiagen, Hilden, Germany). Complementary DNA (cDNA) was prepared using a PrimeScript™ RT Reagent Kit (TaKaRa, Kusatsu, Japan), according to the manufacturer's instruction. Real-Time PCR was performed in a Light Cycler 480 II Real-Time PCR system (Roche Diagnostics, Basel, Switzerland) using SYBR® Green (TaKaRa, Kusatsu, Japan). The sequences of the primers were shown in Table S1. The mRNA level was quantitated by the 2^-ΔΔCt^ method and normalized to the level of GAPDH mRNA.

### NAD^+^/NADH measurements

NAD^+^/NADH level was assayed with the NAD^+^/NADH assay kit (Bioassay Systems, Hayward, CA). 3T3-L1 cells treated differently were homogenized with either 100 μL NAD^+^ extract buffer or NADH extract buffer. The supernatant was uesd for NAD^+^ or NADH assays according to the protocol, then the ratio of NAD^+^/NADH was calculated.

### Statistical analysis

All statistical analyses were performed using SPSS, version 16.0 (SPSS Inc., IL, USA). In experiments, Student's t-test was used to compare differences between two groups. To compare differences between multiple groups, one-way analysis of variance (ANOVA) was used.* P*-values<0.05 was considered as statistically significant.

## Results

### Berberine significantly suppresses high-fat induced weight gain by increasing energy metabolism

**Figure 1:**
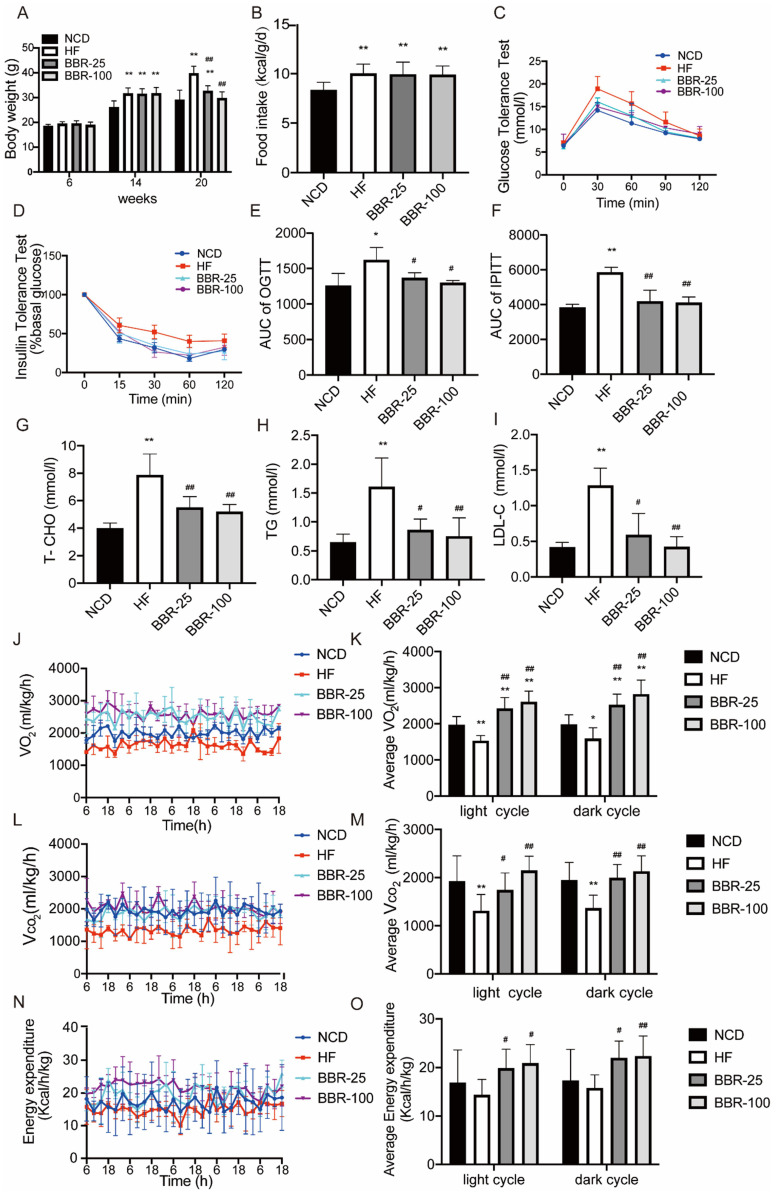
** BBR significantly suppresses high-fat induced weight gain by increasing energy metabolism.** Male C57BL/6J mice aged 6 weeks were randomly divided into four groups as NCD, HF, BBR-25, BBR-100 group. Mice in HF, BBR-25, and BBR-100 were fed by high-fat diet from 6 weeks. After 8 weeks, the HF, BBR-25, and BBR-100 group were respectively treated with 0.9% sterile saline, BBR (25 mgkg-1), and BBR (100 mgkg-1) by gavage until 20 weeks of age. (A) Body weight. (B) Food intake after BBR treatment. (C) Blood glucose levels during the oral glucose tolerance test (OGTT) after BBR treatment 5 weeks. (D) Blood glucose levels during the intraperitoneal injection of insulin tolerance test (IPITT) after BBR treatment 5 weeks. (E) The areas under curve (AUC) of OGTT. (F) AUC of IPITT. (G-I) The values of biochemical indicators including total cholesterol (T-CHO), triglyceride (TG), and low-density lipoprotein cholesterol (LDL-C) were measured in each group after 12 h fasting at BBR treatment 6th week. The data are presented as the mean± SEM (n=10), ^*^P<0.05 and ^**^P<0.01 compared with NCD, ^#^*P*<0.05 and ^##^*P*<0.01 compared with HF. A 5-day indirect calorimetry study was performed using metabolic cages after a 2-day acclimation. The rate of (J-K) V_O2_, (L-M) V_CO2_, and (N-O) energy expenditure were measured in each group after 8 weeks treatment under basal condition, ^*^P<0.05 and ^**^P<0.01 compared with NCD, ^#^*P*<0.05 and^ ##^*P*<0.01 compared with HF.

To evaluate the effects of BBR on energy metabolism, the body weight of each group respectively treated with NCD, HF, and different doses of BBR was determined (Figure 1A). These results showed at 20 weeks the body weight significantly increased in HF group compared with NCD group (*P*<0.01), whereas BBR significantly suppressed body weight of mice with a high-fat diet in a dose-dependent manner (*P*<0.01). The imbalance of energy intake and consumption causes the fluctuations of body weight, so we analyzed the energy intake after the BBR treatment. The results showed that the energy intakes of high-fat diet mice with or without BBR treatment were all higher than that of NCD group (*P*<0.05), but there was no significant difference among HF group and HF with different doses of BBR groups (Figure.1B). Thus, the increase of energy expenditure may be the key factor for BBR to affect the body weight in high-fat diet mice. Moreover, excessive accumulation of WAT is always associated with insulin resistance and dyslipidemia. OGTT and IPITT were performed to evaluate insulin sensitivity*.* Compared to NCD group, HF group had higher blood glucose concentrations after intervention with glucose or insulin (*P*<0.05). Both BBR-25 and BBR-100 group exhibited lower blood glucose concentrations than HF group in OGTT and IPITT (*P*<0.05), which indicated that BBR can elicit beneficial metabolic effects in high-fat diet mice(Figure 1C-F). Additionally, it can also be found that BBR could protect plasma total cholesterol, triglyceride, and low-density lipoprotein cholesterol from dyslipidemia caused by high-fat diet (Figure 1G-I). Metabolic cage data were obtained during the light and dark cycles for 5 days following over 2 days of acclimatization (Figure 1J-O). Our results showed that V_O2_, V_CO2_, and energy expenditure were higher in the groups treated with BBR than in HF group (*P*<0.05). It is consistent with our hypothesis stating that BBR significantly suppresses high-fat induced weight gain by increasing energy metabolism.

### Berberine increases the content of brown adipose tissue and promotes the emergence of characteristics of adipose tissue browning in HF mice

**Figure 2:**
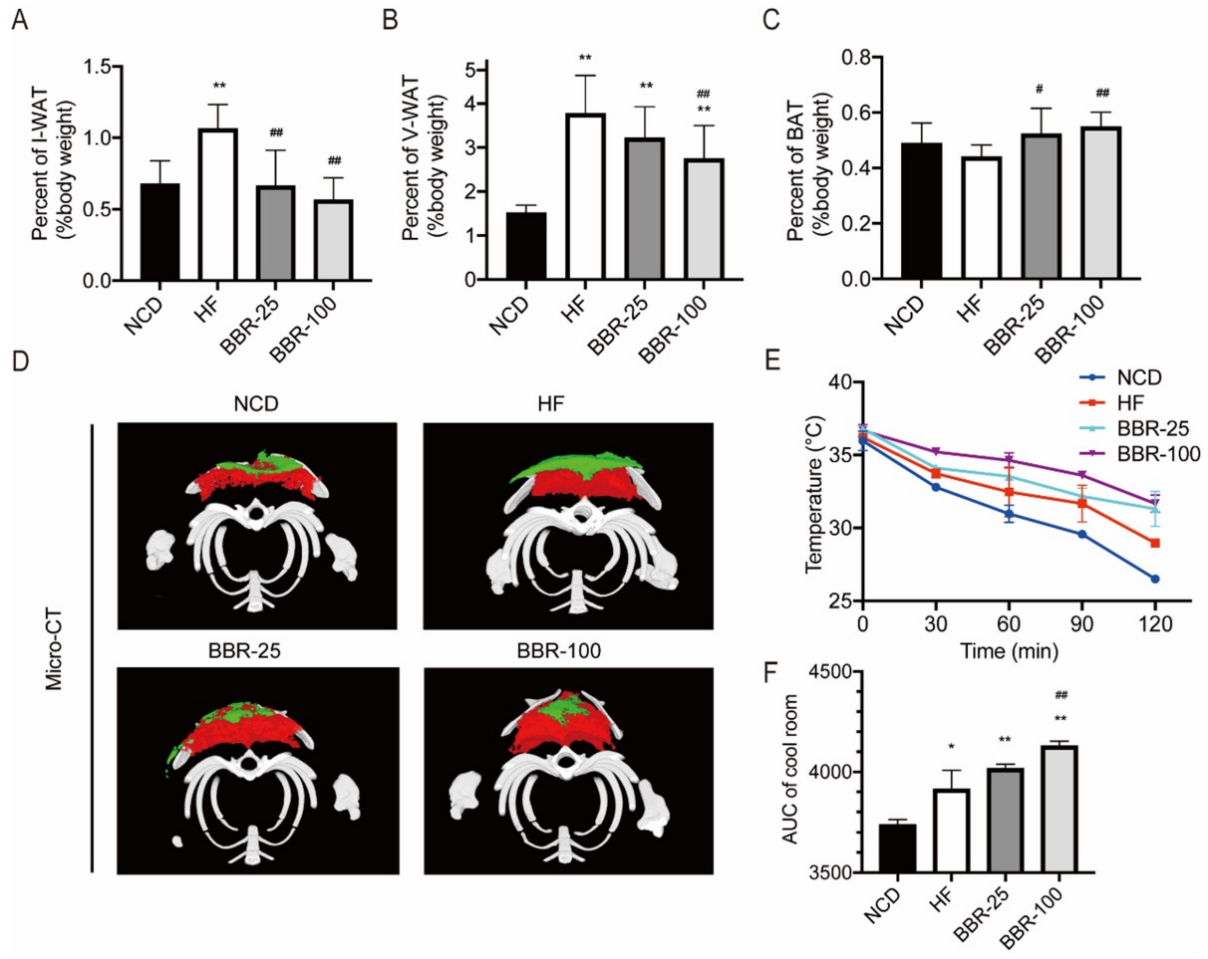
** BBR increases the content of BAT and promotes the emergence of characteristics of adipose tissue browning in HF mice.** The ratio of (A) inguinal white adipose tissue (I-WAT) (B) visceral white adipose tissue (V-WAT), and (C) brown adipose tissue (BAT) to total body weight, values were expressed as means± SEM (n=10), ^*^P<0.05 and ^**^P<0.01 compared with NCD, ^#^*P*<0.05 and ^##^*P*<0.01 compared with HF. (D) Micro-CT scanning and 3D reconstruction of scapular area of mice in each group. Red areas represent interscapular BAT, Green areas represent interscapular WAT. (E) The core temperatures and (F) AUC of core temperature levels under cool exposure test were recorded every 30 min at 4°C for 2 h. Values were expressed as means± SEM (n=10), ^*^P<0.05 and ^**^P<0.01 compared with NCD, ^#^*P*<0.05 and ^##^*P*<0.01 compared with HF.

In mammals, adipose tissue plays a major role in regulating whole-body energy expenditure^41^. Inguinal white adipose tissue (I-WAT), visceral white adipose tissue (V-WAT), and BAT of mice sacrificed at 20 weeks were isolated and weighed. The ratios of I-WAT and V-WAT to body weight increased significantly in HF group than in NCD group (*P*<0.01). When HF mice received BBR treatment, the I-WAT/body weight ratio dropped back to nearly the NCD level (Figure 2A). The V-WAT/body weight ratio decreased significantly in BBR-100 group than in HF group (*P*<0.01), while it was still higher than that of NCD group (Figure 2B). There was no significant difference in V-WAT/body weight ratio between BBR-25 group and HF group, and both of them were significantly higher than NCD group (*P*<0.01). With BBR treatment, the BAT/body weight ratio was significantly higher than that of HF group (*P*<0.05) (Figure 2C). To observe the distribution of adipose tissue more intuitively, Micro-CT scan and 3D reconstruction of the interscapular region were performed. These results further confirmed that BBR increased the content of BAT and reduced the content of subcutaneous WAT (Figure 2D). Adipose tissue browning is characterized by adaptive thermogenesis to maintain core temperature under cold conditions^42^. In cool exposure test, BBR-100 group exhibited outstanding ability of adaptive thermoregulation than HF group (Figure 2E, F). The above experiments demonstrate that BBR increases the content of BAT, decreases the content of WAT, and increases adaptive thermogenesis in high-fat diet mice.

### Berberine promotes the thermogenesis in white adipose tissue and brown adipose tissue of HF mice

Browning WAT tends to exhibit morphological and functional characteristics of BAT. Brown adipocytes have multiple small lipid droplets, as opposed to white adipocytes that have a single massive lipid droplet. In HE staining, the lipid droplets of the BAT and I-WAT both showed smaller in BBR treated groups than HF group dose-dependently. The expression of UCP-1 which is an indicator of the thermogenic capacity in adipocytes can be used to differentiate brown adipocytes from white adipocytes^43^. Immunohistochemical analysis of adipose tissues revealed that the expressions of UCP1 in I-WAT and BAT significantly increased in BBR treated groups than HF group. Consistent with the results of immunohistochemical analysis, Western blot showed that UCP1 expressions significantly increased in IWAT and BAT of BBR treated groups than HF group. The mRNA levels of thermogenesis genes and fatty acid oxidation genes in I-WAT and BAT of mice treated with BBR were significantly higher than those in mice treated with HF alone (*P*<0.05). In the above experiments, we furtherly demonstrate that BBR promotes the WAT browning and the thermogenic activity of BAT in HF mice.

### Berberine promotes remodeling and thermogenesis of adipose tissue via SIRT1- deacetylated PPARγ

**Figure 4:**
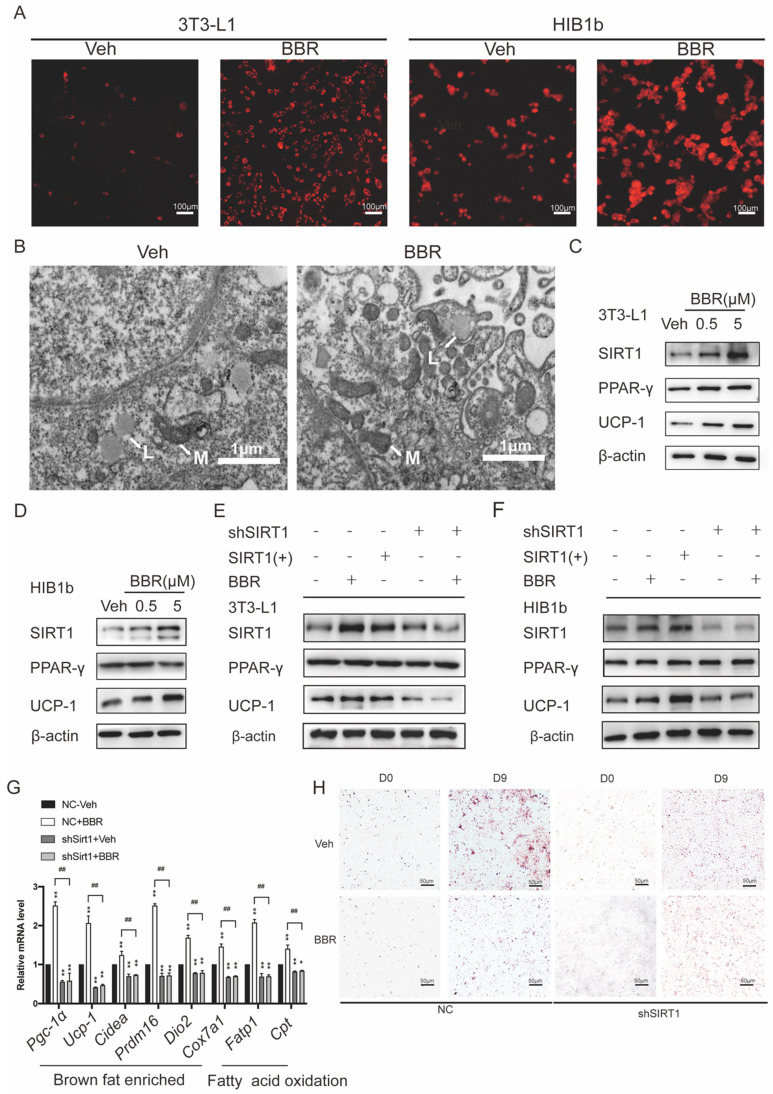
** The regulation of adipose tissue remodeling and thermogenesis by BBR is dependent on SIRT1.** After 8 days of differentiation, 3T3-L1 and HIB1b cells were treated with vehicle or BBR (5 μM) for 24 h. (A) The fully differentiated cells were fixed and subjected to Mito-tracker Red staining, Scale bar=100μm. (B) Representative transmission electron microscopy images from fully differentiated 3T3-L1 cells treated with vehicle and BBR (5 μM), Scale bar=1μm (×12000). L: lipid droplets, M: mitochondria. The proteins of SIRT1, PPARγ, and UCP1 were expressed in fully differentiated (C) 3T3-L1 and (D) HIB1b cells treated with vehicle or BBR (0.5 μM and 5 μM, respectively) for 24 h. 3T3-L1 and HIB1b cells with stable knockdown SIRT1 were established by lentivirus transfection. (E) 3T3-L1 and (F) HIB1b cells transfected with SIRT1 plasmid for 36 h or treated with BBR for 24 h. SIRT1 knockdown 3T3-L1 cells were treated with or without BBR for 24 h. The expressions of SIRT1, PPARγ, and UCP1 were detected by Western blot. (G) Relative mRNA levels of thermogenesis genes and fatty acid oxidation genes in NC or SIRT1 knockdown 3T3-L1 cells after differentiation treated with vehicle or BBR 12 h. ^*^P<0.05 and ^**^P<0.01 compared with NC-Veh, ^##^*P*<0.01 compared with NC-BBR. (H) After full differentiation, 3T3-L1 and *shSIRT1* 3T3-L1cells were treated with or without BBR for 24 h and stained Oil red O.

3T3-L1 white preadipocytes and HIB1b brown preadipocytes were utilized to assess the ability of BBR to promote brown remodeling and thermogenesis *in vitro.* 3T3-L1 and HIB1b cells were incubated with Mito-Tracker Red. It can be observed that BBR treated adipocytes showed obviously strong red stain in the cytoplasm, indicating that there were more mitochondrion (Figure 4A). Transmission electron microscopy showed that 3T3-L1 cells treated with BBR exhibited the characteristics of brown-like adipocytes with increased mitochondrial density and smaller lipid droplets (Figure 4B). Previous studies have demonstrated that SIRT1 played an important role in energy metabolism and remodeling of adipose tissue^21^, ^44^, ^45^. We next elucidated whether SIRT1 is necessary for BBR to regulate the remodeling of adipose tissue. Consistent with the data in mice, Western blot showed the expressions of SIRT1 and UCP1 in 3T3-L1 and HIB1b cells with BBR treatment were increased in a dose-dependent manner, but not PPARγ (Figure 4C, D). BBR treatment and SIRT1 overexpression both significantly increased the UCP1 expression. However, when the expressions of SIRT1 in 3T3-L1 and HIB1b cells were blocked by lentivirus, the total increased expression of UCP1 induced by BBR was prevented (Figure 4E, F). The mRNA levels of thermogenesis genes and fatty acid oxidation genes were increased by BBR (*P*<0.05), but not in sh*SIRT1* cells, indicating that BBR-induced brown remodeling was dampened by SIRT1 knockdown (Figure 4G). Strikingly, Oil Red O staining revealed that the reduction of lipid accumulation in white adipocytes caused by BBR was blocked in 3T3-L1 cell lines stably expressing shRNA-targeting SIRT1 (Figure 4H). Taken together, these data suggest that BBR is dependent on SIRT1 for modulating brown remodeling of adipocytes.

**Figure 5:**
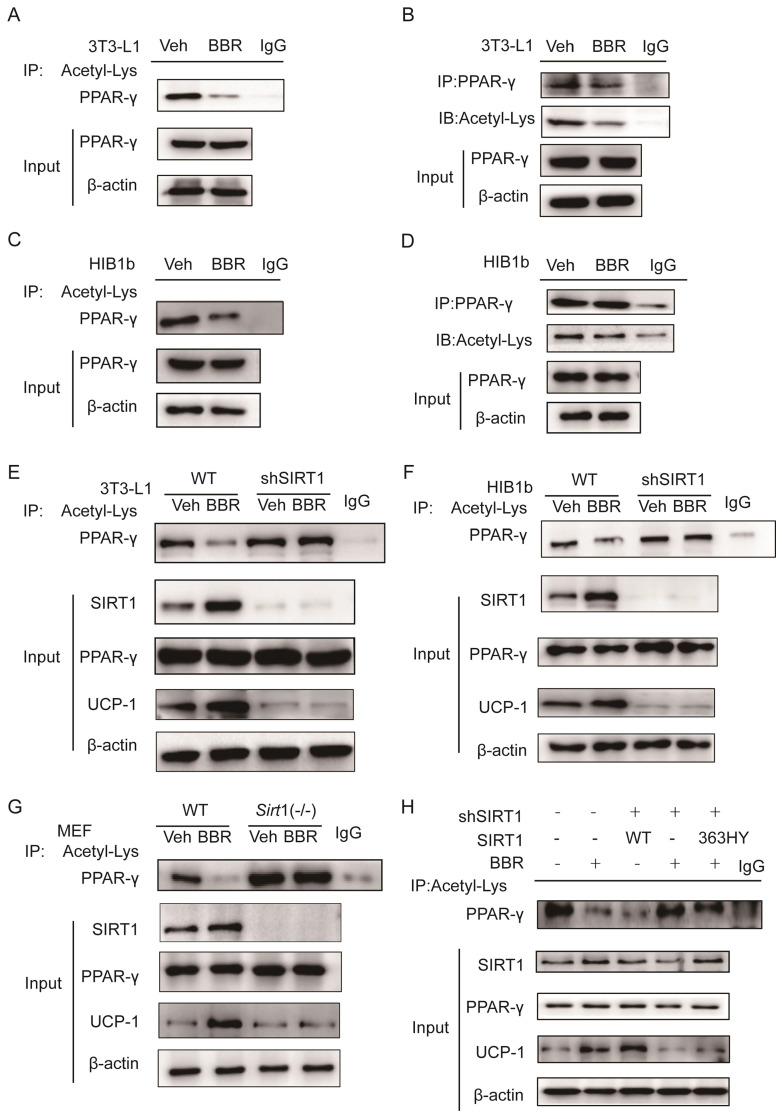
** BBR increases the deacetylated level of PPARγ by SIRT1 activation.** The levels of PPARγ acetylation were measured in fully differentiated (A, B) 3T3-L1 and (C, D) HIB1b cells treated with BBR (5 μM) for 24 h. The levels of PPARγ acetylation were examined via immunoprecipitation (IP) with anti-acetylated lysine antibody and anti-PPARγ antibody. The effects of BBR on PPARγ acetylation were determined in WT and SIRT1 knockout (E) 3T3-L1 and (F) HIB1b cells, respectively. (G) WT and *Sirt*1^-/-^ mouse embryonic fibroblasts (MEFs) were treated with or without BBR (5 μM) for 24 h. (H) Flag-SIRT1 plasmid or catalytically inactive mutant 363 HY plasmid was transfected into stably SIRT1 knockdown 3T3-L1 cells. WT and SIRT1 knockdown 3T3-L1 cells were treated with BBR (5 μM) for 24 h. The levels of PPARγ acetylation were examined by IP with anti-acetylated lysine antibody.

**Figure 6:**
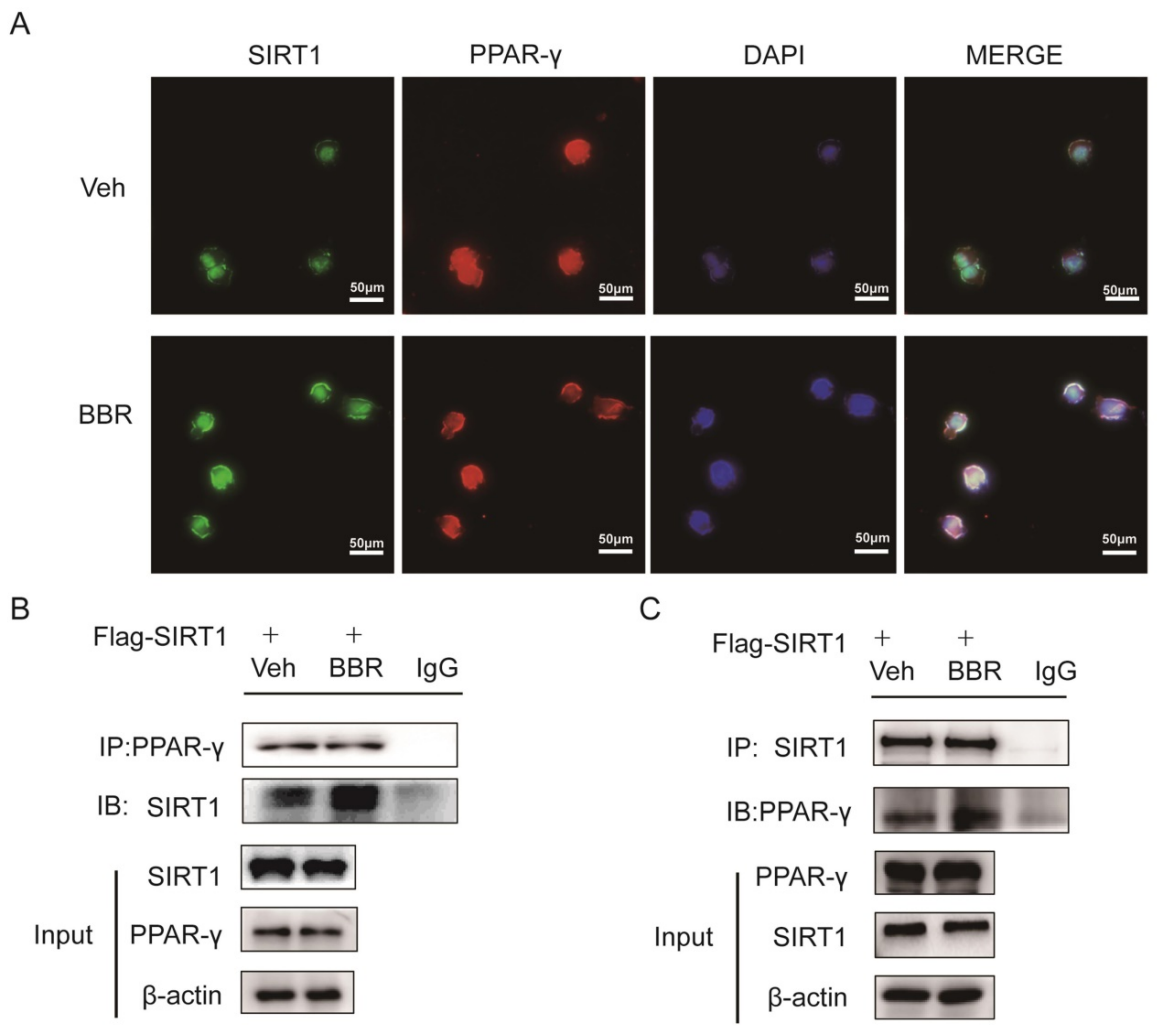
** BBR promotes the interaction between SIRT1 and PPARγ.** (A) Representative immunofluorescence images demonstrating colocalization of SIRT1 (green) and PPARγ (red) in differentiated 3T3-L1 cells treated with BBR (5 μM) for 24 h. (B, C) 3T3-L1 cells were transfected with Flag-SIRT1 plasmid for 36 h and treated with vehicle or BBR (5 μM). The expressions of SIRT1 and PPARγ were respectively examined by IP with anti-PPARγ antibody and anti-SIRT1 antibody.

Previous studies demonstrated that SIRT1 can regulate the browning of adipose tissue by adjusting the acetylation level of PPARγ^21^. In this study, it has been confirmed that the total amount of PPARγ in adipose tissue did not change significantly after BBR treatment. Therefore, we hypothesized the effect of BBR on brown remodeling in adipose tissue may be based on the adjustment of the level of PPARγ acetylation. To verify our speculation, 3T3-L1 and HIB1b cells were treated with BBR after differentiation, and then analyzed for the level of PPARγ acetylation via IP assay with anti-acetylated lysine antibody and anti-PPARγ antibody. The results showed that after BBR treatment, the levels of PPARγ acetylation in 3T3-L1 and HIB1b cells were significantly decreased (Figure 5A-D), indicating BBR increased the deacetylation modification of PPARγ. To further elucidate whether SIRT1 is the deacetylase that is responsible for PPARγ deacetylation, wild type and stably knocking down SIRT1 of 3T3-L1 and HIB1b cells were respectively treated by BBR. It can be found that SIRT1 knockdown resulted in the disappearance of the changes in acetylation level of PPARγ induced by BBR treatment (Figure 5E-F). *Sirt1*^-/-^MEF cells further corroborated this idea (Figure 5G), suggesting that increased deacetylation modification of PPARγ by BBR depended on SIRT1 activation. Then we further transfected WT-SIRT1, *shSIRT1*, and SIRT1 mutant (363HY) plasmids into 3T3-L1 cells treated with BBR. It can be inferred from the results that BBR significantly increased the PPARγ deacetylation by WT-*Sirt1* plasmid, but not catalytically inactive *Sirt1* mutant (H363Y) plasmid (Figure 5H). Immunofluorescence revealed that SIRT1 and PPARγ were intensively colocalized in 3T3-L1 cells treated with BBR (Figure 6A). In 3T3-L1 cells with SIRT1 overexpression, we detected SIRT1 and PPARγ respectively in PPARγ and SIRT1 immunoprecipitates from cell lysates increased in cells with BBR treatment, which further proved that BBR caused more binding of SIRT1 and PPARγ (Figure 6B-C). The above experiments demonstrate that BBR increases the deacetylated level of PPARγ by promoting the interaction between SIRT1 and PPARγ.

### Berberine affects SIRT1 protein levels and activity to regulate PPARγ deacetylation levels in an AMPK-dependent manner

**Figure 7:**
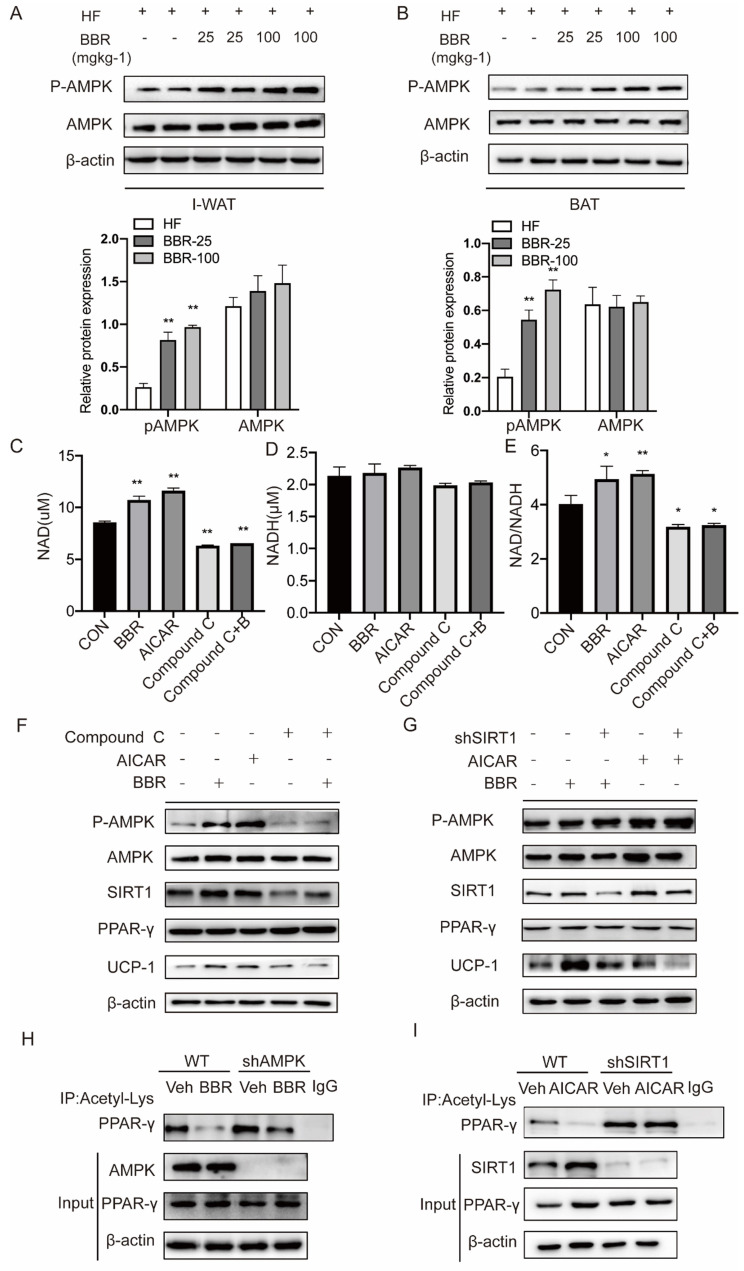
** Berberine affects SIRT1 protein levels and activity to regulate PPARγ deacetylation levels in an AMPK-dependent manner.** Western blot analyzes the expressions of P-AMPK and total-AMPK in (A) I-WAT and (B) BAT of HF mice treated with vehicle or BBR (25 mgkg^-1^, 100 mgkg^-1^). (C-E) 3T3-L1 cells were pretreated with BBR (5μM), AMPK activator AICAR (0.5mM), AMPK inhibitor Compound C (20μM), and Compound C+ BBR (20μM) for 4h. The levels of NAD+ and NADH were measured with the kits, and their ratios were calculated. (F)3T3-L1 cells were treated with AICAR (0.5 mM), Compound C (20 μM), and BBR (5 μM) for 12 h. (G) WT and SIRT1 knockdown 3T3-L1 cells were treated with BBR (5 μM) or AICAR (0.5 mM) for 12 h. Representative proteins were detected by Western blot. (H) 3T3-L1 cells with stable knockdown AMPK were established using lentivirus transfection. WT and AMPK knockdown 3T3-L1 cells were treated with vehicle or BBR (5 μM) for 12 h. (I) WT and SIRT1 knockdown 3T3-L1 cells were treated with vehicle or BBR (5 μM) for 12 h. The levels of PPARγ acetylation were examined by IP with anti-acetylated lysine antibody.

AMPK plays a key role in the thermogenic program induced by BBR, but the mechanism is not clear^46^. We examined the expressions of AMPK in response to BBR treatment in IWAT and BAT by Western blot. The results showed the P-AMPK expressions in IWAT and BAT of mice with BBR treatment were increased in a dose-dependent manner than HF group, but not AMPK. This indicates that BBR can activate AMPK both in subcutaneous WAT and BAT (Figure 7A-B). AMPK has been reported to activate SIRT1 by modulating the NAD^+^/NADH ratio^33^. Since SIRT1 deacetylase activity is driven by the NAD^+^/NADH ratio^47^, we hypothesized that BBR activates AMPK to change the intracellular NAD^+^/NADH ratio to activate SIRT1.To support this hypothesis, we treated 3T3-L1 cells with BBR, AICAR (AMPK activator), Compound C (AMPK inhibitor) and Compound C+BBR pretreated 3T3-L1 cells. It was found that BBR and AICAR increased the NAD^+^ and NAD^+^/NADH ratio in 3T3-L1 cells and the effect of BBR was disappeared after the co-treatment with Compound C (Figure 7C-E). In this study, we also can observe that BBR and AICAR increased the expressions of UCP1. Compound C diminished the BBR-induced expressions of UCP1 (Figure 7F). The expressions of UCP1 induced by BBR and AICAR were significantly attenuated by SIRT1 knockdown (Figure 7G). To further elucidate whether BBR regulates PPARγ deacetylation via AMPK, we constructed 3T3-L1 cell line with knockdown AMPK expression using lentivirus. Wild-type and shAMPK 3T3-L1 cells were treated with BBR. It can be found that the change in acetylation level of PPARγ induced by BBR was significantly blocked in shAMPK 3T3-L1 cells (Figure 7H). Moreover, in the *shSIRT1* 3T3-L1 cells, PPARγ deacetylation induced by AICAR was found to be inhibited (Figure 7I). From these, we conclude that AMPK regulates the level of PPARγ deacetylation in a SIRT1-dependent manner.

## Discussion

Obesity is a status of chronic positive energy balance associated with excess fat storage in adipose tissues^48^. The conversion of fat-accumulating WAT into energy-dissipating BAT may be an effective and potentially harmless solution. Cold exposure and β-adrenergic receptor agonists are the most effective approaches to induce browning of adipose tissue^49^. However, considering the risks and side effects, it is necessary to develop new therapeutic strategies for obesity by remodeling of adipose tissue in the normal state. BBR has the potential efficacy in correcting abnormal lipid metabolism and increasing metabolic rate, while its effects and mechanisms in simultaneously regulating WAT and BAT have not been fully elucidated. In this study, we constructed a model of high-fat feeding-induced obesity, and gave the lowest effective concentration and higher concentration of BBR intervention to analyze the effect of BBR on adipose tissue. Meanwhile, we used 3T3-L1 white preadipocytes and HIB1b brown preadipocytes to further investigate the potential mechanism. The following conclusions are drawn from this study: 1) BBR enhances the browning of WAT and the thermogenesis of BAT, thereby reducing weight gain and obesity-related abnormalities of glucolipid metabolism in high-fat diet mice. 2) BBR enhances the AMPK/SIRT1 pathway to selectively activate PPARγ, thereby stimulating adipose tissue browning and thermogenesis.

BBR is proven to regulate glucolipid metabolism and attenuate insulin resistance^25^, ^50^, ^51^. Adipose tissue dysfunction is the key to obesity and its complications. Therefore, researchers have focused on the effects of BBR on adipose tissue in recent years. Many studies suggested that BBR not only prevents obesity through downregulating the expressions of the genes that promote the proliferation and differentiation of adipocytes, but it also inhibits the accumulation of adipose tissue by activating the enzymes associated with the uptake of glucose and fatty acids^52^, ^53^. Moreover, BBR can attenuate adipose tissue fibrosis induced by high-fat diet and reduce the inflammatory response in adipose tissue, thereby improving insulin resistance^54^, ^55^. In type 2 diabetic mice, BBR has been reported to promote glucose utilization and reduce triglyceride uptake and synthesis by increasing the expressions of liver X receptors (LXRs) and peroxisome proliferator-activated receptors (PPARs) and decreasing the expressions of sterol regulatory element-binding proteins (SREBPs) in WAT^56^. However, the effects of BBR on the energy expenditure of WAT and BAT are still controversial. It has been suggested that BBR can improve energy expenditure by increasing adipose tissue thermogenesis^25^. This conclusion is consistent with our findings. This study has further confirmed that BBR enhanced energy expenditure of high-fat diet mice in a dose-dependent manner. Another study has shown that BBR exerted promotional effects on energy expenditure mainly due to its ability to increase the content of BAT, but it has no significant influence on WAT^57^. However, our study found that BBR increased the content of BAT and promoted the browning of subcutaneous WAT. This may be attributed to the adipocytes from different sources employed in our study. There are two main ways for browning of WAT, including direct differentiation from adipose precursor cells and transdifferentiation from existing white adipocytes. In the present study, we performed BBR intervention on mature white adipocytes rather than SVF cells isolated from WAT. Therefore, BBR may induce the browning of subcutaneous white adipocytes mainly by acting on mature white adipocytes, rather than by affecting the differentiation of precursor cells.

We further investigated the mechanism by which BBR regulates adipose tissue remodeling. PPARγ is specifically highly expressed in adipose tissue, and is closely associated with adipose tissue differentiation and remodeling. Recently, many studies have been carried out about the effect of BBR on PPARγ in adipose tissue, but their conclusions are inconsistent. Some suggested that BBR may inhibit the expression of PPARγ, thereby impeding the differentiation of adipose tissue and reducing their formation^58^. However, other studies showed a significant increase in the expression of PPARγ in adipose tissue following BBR treatment, which promotes the formation of BAT and enhances fatty acid oxidation^55^, ^59^. Moreover, it was also reported that BBR has no significant effect on the expression of PPARγ^53^. In our study, we have found that the administration of BBR in mature adipocytes selectively activated PPARγ mainly by modulating the level of PPARγ acetylation. The selective transcription of PPARγ in WAT and BAT is dependent on deacetylation induced by SIRT1. Deacetylation of Lys268 and Lys293 on PPARγ induced by SIRT1 recruits the BAT program coactivator Prdm16, leading to the selective induction of BAT genes and repression of WAT genes associated with insulin resistance. Relatively, acetylation of Lys268 and Lys293 on PPARγ promotes transcription of lipid synthesis related genes^21^. Previous studies demonstrated that PPARγ can improve insulin sensitivity by inducing adipose tissue browning, which helps to reduce the occurrence of metabolic related diseases^60^. Although changes in the total amount of PPARγ may cause damage to target organs, which greatly limits its clinical application, selective activation of PPARγ provides a potential mechanism for the treatment of obesity and related diseases^14^, ^16^-^18^. In our study, we have analyzed white adipocytes, brown adipocytes, and embryonic fibroblasts, thus demonstrated that BBR could act as a SIRT1-dependent selective PPARγ activator.

BBR has been considered as an AMPK activator. The mechanism of AMPK activation by BBR may be a rise in the AMP/ ATP^31^. AMPK, as a crucial cellular energy sensor, can regulate whole-body nutrient metabolism^61^. BBR can induce activation of the AMPK pathway in adipose tissue, leading to increasing glucose uptake and attenuating insulin resistance^29^, ^30^. In addition, BBR was confirmed to alleviate adipose tissue fibrosis in high-fat diet mice by activating the AMPK pathway^54^. Recently, several studies have identified that AMPK plays a key role in BBR-induced thermogenesis of adipose tissue^25^. However, the molecular mechanism underlying remains unclear. Our study demonstrated that the acetylation level of PPARγ was reduced by the administration of AICAR and BBR in adipocytes. Notably, the regulation of the acetylation level of PPARγ by BBR was partially counteracted in adipocytes with knockdown of AMPK. Therefore, it can be inferred that BBR selectively regulates the acetylation level of PPARγ through activation of AMPK. There is growing evidence indicating that SIRT1 has regulatory effects in glucose homeostasis, insulin sensitivity, and adipogenesis via its deacetylase activity^32^. BBR inhibits lipid accumulation in hepatocytes and enhances mitochondrial function in muscle in a SIRT1-dependent manner^62^, ^63^. BBR can also exert antioxidant activity and ameliorate atherosclerosis by upregulating SIRT1^64^. Previously it was established that BBR can upregulate SIRT1 expression to suppress inflammatory response and alleviate insulin resistance in adipose tissue^55^, ^65^. There is a complex interaction between AMPK and SIRT1, which allows them to exert similar functions in cellular metabolism, inflammation, and mitochondrial function. AMPK-dependent phosphorylation and nuclear translocation of GAPDH supply a fast track for SIRT1 activation^66^. AMPK can also enhance the activity of SIRT1 by increasing cellular NAD^+^/NADH levels, resulting in deacetylating downstream SIRT1 targets and modulating their activity^33^. In our study, we also found that BBR increased the NAD^+^/NADH ratio in 3T3-L1 cells dependent on AMPK. AMPK was also linked to an increase in SIRT1 protein levels^67^. In turn, SIRT1 can deacetylate serine/threonine kinase liver kinase B1 (LKB1) to activate AMPK^68^. The effects of SIRT1 and AMPK can also be amplified by a mutual positive regulatory loop. AMPK may function together with SIRT1 in mitochondrial biogenesis. In this study, it can be observed that the regulation of PPARγ deacetylation by AICAR and BBR was eliminated in adipocytes with knockdown of SIRT1, indicating that the regulation of PPARγ deacetylation by BBR-activated AMPK is dependent on SIRT1. Thus, we can infer that selective activation of PPARγ by BBR stimulates adipose tissue browning and thermogenesis by enhancing the AMPK/SIRT1 signaling pathway, which offers a novel insight into the regulation of adipose tissue by BBR.

In conclusion, we further confirm that BBR promotes the thermogenic activity of BAT and browning of WAT, thereby alleviating high-fat diet induced weight gain and abnormal glucolipid metabolism. Furthermore, BBR increases the level of deacetylation of PPARγ through activating the AMPK/SIRT1 pathway, which promotes adipose tissue remodeling and thermogenesis. It is suggested that BBR can act as a selective PPARγ activator and is expected to be a new therapeutic strategy for obesity and metabolic diseases. This work provides a new perspective for the further clinical application of BBR.

## Supplementary Material

Supplementary table.

## Figures and Tables

**Figure 3:**
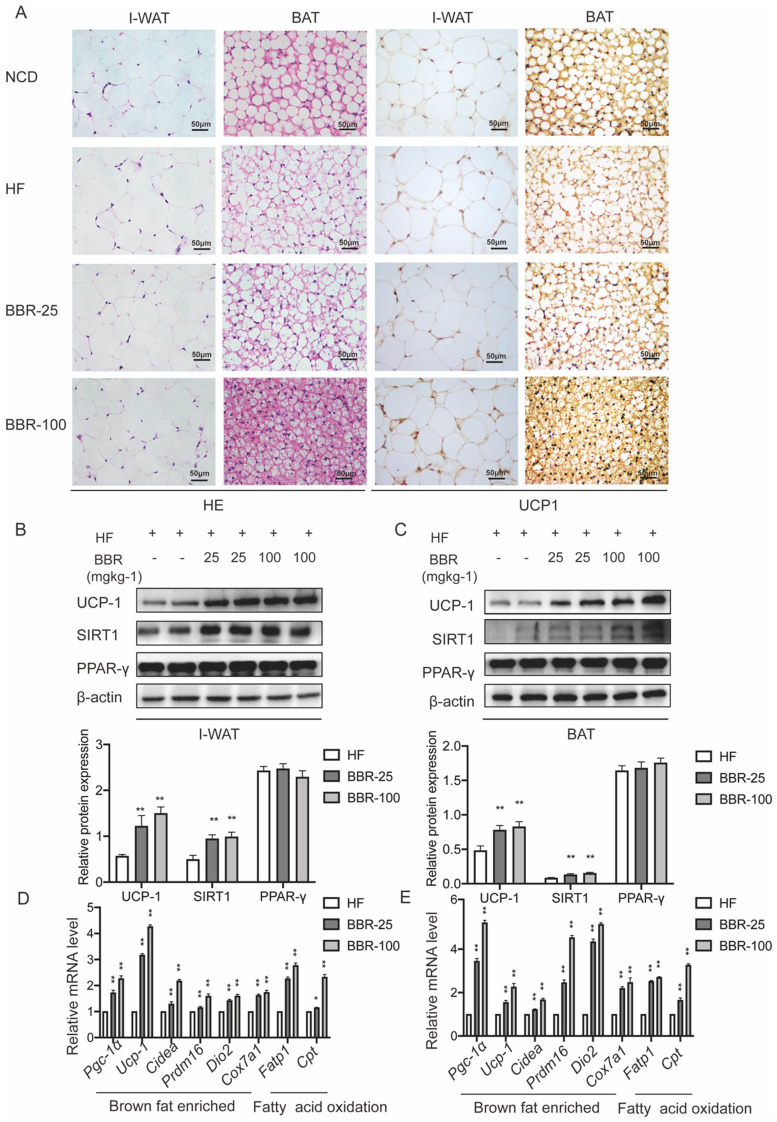
** BBR promotes the thermogenesis in WAT and BAT of HF mice.** (A) Representative HE staining and immunohistochemistry staining of UCP1 (yellow) in I-WAT and BAT of mice, Scale bar=50 μm. Western blot analyzes the key protein changes in (B) I-WAT and (C) BAT of HF mice treated with vehicle or BBR (25 mgkg^-1^, 100 mgkg^-1^). Relative mRNA levels of thermogenesis and fatty acid oxidation gene in (D) I-WAT and (E) BAT of HF mice after BBR treatment. Values were expressed as means± SEM (n=10), ^*^P<0.05 and ^**^P<0.01 compared with HF.

## Abbreviations

BBR: berberine
WAT: white adipose tissue
BAT: brown adipose tissue
NCD: normal-chow diet
UCP1: uncoupling protein 1
T-CHO: total cholesterol
TG: triglyceride
LDL-C: low-density lipoprotein cholesterol
AMPK: AMP-activated protein kinase
P-AMPK: phosphorylation-AMPK
SIRT1: Sirtuin 1
PPARγ: peroxisome proliferator-activated receptors-gamma
