# Supplementary material for: Berberine modulates deacetylation of PPARγ to promote adipose tissue remodeling and thermogenesis via AMPK/SIRT1 pathway
Source: Int J Biol Sci. 2021 Jul 25;17(12):3173–87. doi: 10.7150/ijbs.62556 (PMC8375237; doi:10.7150/ijbs.62556)
Supplement: Supplementary file 1 — Supplementary table. [file ijbsv17p3173s1.pdf]

Supplementary Materials for

Berberine modulates deacetylation of PPARγ to promote adipose tissue remodeling and thermogenesis via AMPK/SIRT1 pathway

Table 1

Primer sequences

Pgc-1α forward	GGATATACTTTACGCAGGTCGA
Pgc-1α reverse	CGTCTGAGTTGGTATCTAGGTC
Ucp1 forward	ATTCAGAGGCAAATCAGCTTTG
Ucp1 reverse	GTGTTTCTCTCCCTGAAGAGAA
Cidea forward	CAATGTCAAAGCCACGATGTAC
Cidea reverse	CTGTGCAGCATAGGACATAAAC
Prdm16 forward	CAACAAAGAGAAGCCGTTCAAG
Prdm16 reverse	TTTCGGATCTCGGAGAAGTAAG
Dio2 forward	AATTATGCCTCGGAGAAGACCG
Dio2 reverse	GGCAGTTGCCTAGTGAAAGGT
Cox7a1 forward	CTCTTCCAGGCCGACAATGA
Cox7a1 reverse	GCCCAGCCCAAGCAGTATAA
Fatp1 forward	CCTCTCTGTTCTGATTCGTGTT
Fatp1 reverse	GTCCAGCATATACCACTACTGG
Cpt forward	CTACATCACCCCAACCCATATT
Cpt reverse	GATCCCAGAAGACGAATAGGTT
